# Newcastle Disease Virus as a Vaccine Vector for 20 Years: A Focus on Maternally Derived Antibody Interference

**DOI:** 10.3390/vaccines8020222

**Published:** 2020-05-14

**Authors:** Zenglei Hu, Jie Ni, Yongzhong Cao, Xiufan Liu

**Affiliations:** 1 Joint International Research Laboratory of Agriculture and Agri-Product Safety, The Ministry of Education of China, Yangzhou University, Yangzhou 225009, China; zengleihu@163.com (Z.H.); yzucao@yzu.edu.cn (Y.C.); 2 Institutes of Agricultural Science and Technology Development, Yangzhou University, Yangzhou 225009, China; 3 Animal Infectious Disease Laboratory, School of Veterinary Medicine, Yangzhou University, Yangzhou 225009, China; jenniezs@163.com; 4 Jiangsu Co-innovation Center for Prevention and Control of Important Animal Infectious Diseases and Zoonoses, Yangzhou University, Yangzhou 225009, China

**Keywords:** Newcastle disease virus, vaccine vector, maternally derived antibody, interference

## Abstract

It has been 20 years since Newcastle disease virus (NDV) was first used as a vector. The past two decades have witnessed remarkable progress in vaccine generation based on the NDV vector and optimization of the vector. Protective antigens of a variety of pathogens have been expressed in the NDV vector to generate novel vaccines for animals and humans, highlighting a great potential of NDV as a vaccine vector. More importantly, the research work also unveils a major problem restraining the NDV vector vaccines in poultry, i.e., the interference from maternally derived antibody (MDA). Although many efforts have been taken to overcome MDA interference, a lack of understanding of the mechanism of vaccination inhibition by MDA in poultry still hinders vaccine improvement. In this review, we outline the history of NDV as a vaccine vector by highlighting some milestones. The recent advances in the development of NDV-vectored vaccines or therapeutics for animals and humans are discussed. Particularly, we focus on the mechanisms and hypotheses of vaccination inhibition by MDA and the efforts to circumvent MDA interference with the NDV vector vaccines. Perspectives to fill the gap of understanding concerning the mechanism of MDA interference in poultry and to improve the NDV vector vaccines are also proposed.

## 1. Introduction

Infectious disease is a major challenge for human beings and animals. With the economic boom and urbanization, infectious diseases keep emerging and re-emerging, causing severe losses for human and animal health. In the 21st century, severe acute respiratory syndrome in 2003 [1], Ebola in 2014 [2] and the latest global pandemic of novel coronavirus disease (COVID-19) in 2019 [3] are only a few examples of devastating emerging infectious diseases. The history of combating infectious diseases proves that vaccination is undoubtedly the most effective means to protect lives from infection. With the progress of immunology, molecular biology and microbiology, the technologies for vaccine development evolve rapidly. In particular, recombinant virus vectors represent a powerful and promising platform to produce safe, immunogenic and efficacious vaccines without cultivating and handling live pathogens, especially those lethal for humans and animals.

Initially, DNA viruses, such as herpesvirus, adenovirus and vaccinia virus, were used as vaccine or gene therapy vectors [4,5,6]. Due to the establishment of reverse genetics, numerous RNA viruses have been explored as delivery vehicles of foreign immunogens [7]. Particularly, Newcastle disease virus (NDV), a non-segmented negative-sense RNA virus (NNSV), belonging to paramyxovirus that naturally infects birds is used as a vector to generate novel vaccines for poultry, mammals, including humans [8,9]. Since generation of the first recombinant NDV expressing a foreign gene in 2000 [10], numerous NDV-vectored vaccines expressing protective antigens from various pathogens have been generated. This period has witnessed a process of cognizing, exploring and optimizing NDV as a vector and also a process of recognizing limitations of this vector. In this paper, we outline the brief history of NDV as a vaccine vector by highlighting some key milestones in this process. We summarize the characteristics of NDV as a vaccine vector as well as the recent advances in the development of novel vaccines and therapeutics based on NDV for poultry and mammals, including humans. More importantly, we focus on the major bottleneck restraining the effectiveness of the NDV vector vaccines in poultry, i.e., the interference of maternally derived antibody (MDA), and discuss the research advances in the mechanisms of vaccination inhibition by MDA and finally present our perspectives for improving the NDV vector.

## 2. Biological Characteristics of NDV as a Vaccine Vector

NDV is a member of the genus *Avulavirus* in the family *Paramyxoviridae*. The genome of NDV is a non-segmented, negative-sense, single-stranded RNA of 15,186, 15,192 or 15,198 nucleotides. The NDV genome is composed of six transcriptional units that encode six main viral proteins, namely nucleocapsid protein (NP), phosphoprotein (P), matrix protein (M), fusion protein (F), hemagglutinin-neuraminidase protein (HN) and large polymerase protein (L) [11]. Additionally, two accessory proteins, V and W, are produced by RNA editing of the P gene. NDV replicates efficiently in vivo and can stimulate a systematic immune response, especially mucosal immunity in the respiratory tract. To summarize, NDV has the following characteristics allowing it to be an ideal vector: (1) the NDV genome is easy to manipulate. The genome is ~15 kb and it is easy to clone the entire genome into a transcriptional plasmid for molecular engineering. (2) High virus yield in chicken embryos. Most lentogenic NDV strains replicate efficiently in chicken embryos and virus yield can reach as high as 9–10 log_10_ in 50% embryo infectious dose (EID_50_) or 9–10 log_2_ in hemagglutination (HA) titer, which allows the large-scale vaccine production. (3) NDV can accommodate and express a foreign gene stably. Consecutive passages of recombinant NDVs in eggs do not affect expression of the transgenes. Next-generation sequencing of a recombinant NDV expressing the glycoprotein D (gD) gene of infectious laryngotracheitis virus (ILTV) after eight serial passages in eggs revealed that none of thirteen single-nucleotide polymorphisms were located in the ILTV gD insert or any critical biological domains [12]. (4) Low risk of gene exchange and recombination. NDV replicates in the cytoplasm and the virus genome does not integrate with the host genome in the nucleus. Moreover, NDV is a NNSV with a much lower frequency of recombination with the host or other microbes. (5) NDV can induce a systematic immune response, including mucosal, humoral and cellular immunity. Lentogenic NDV strains primarily replicate in the respiratory tract and elicit robust local mucosal immunity and subsequently humoral and cellular immunity. (6) NDV vaccines can be administered by mass vaccination approaches. In the field, live NDV vaccines are usually administrated by spraying, drinking water and automatic in ovo injection, which can fulfill the requirement of industrial processes in poultry settings. (7) No pre-existing immunity against NDV in mammals, including humans. NDV is highly host-restricted and infects birds naturally. There is no NDV-specific pre-existing immunity in mammals, including humans, which in turn becomes an advantage of NDV-vectored vaccines in these hosts.

## 3. A Brief History of NDV as a Vector

**Figure 1:**
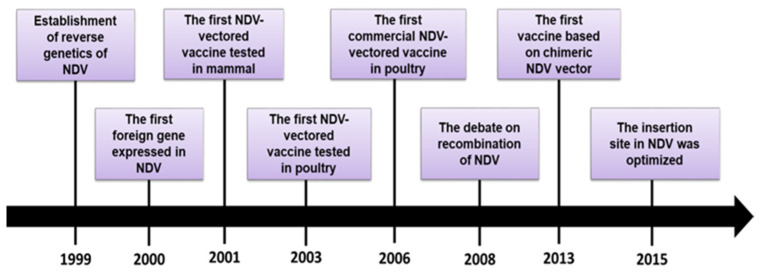
The milestones in the history of Newcastle disease virus as a vaccine vector.

Establishment of reverse genetics of NDV initiated the exploration of the virus as a vector. During the past 20 years, a variety of foreign genes have been expressed in the NDV backbone and the knowledge about the safety, insertion site of foreign genes and vector optimization has grown drastically. In this section, the history is delineated by highlighting some key milestones from our point of view (Figure 1).

In 1999, Peeters et al. constructed a transcription plasmid containing the full-length cDNA clone of NDV La Sota strain as well as three supporting plasmids encoding the NP, P and L proteins [13]. Infectious NDV was successfully rescued for the first time by co-transfecting these four plasmids into the cells. They also demonstrated that the cleavage site of the F protein is the major determinant for NDV virulence through mutating the amino acids in this motif. Later, another research group also reported the successful generation of NDV Clone-30 strain using reverse genetics techniques [14]. These two studies open the era of reverse genetics of NDV and this technique has been established in laboratories worldwide, which remarkably promotes the understanding of the function of viral genes, the molecular basis for virulence and the development of novel vaccines.

One year later, a mesogenic NDV strain Beaudette C (BC) was recovered and more importantly, a recombinant BC strain expressing the foreign gene encoding chloramphenicol acetyltransferase (CAT) was also generated [10]. The CAT gene was expressed between the HN and L genes as an additional independent transcriptional unit (ITU) and the insertion of the foreign gene resulted in growth retardation and attenuation of the recombinant virus. This study was the first proof-of-concept study on expressing a foreign gene in NDV genome and unveiled the potential of NDV as a vaccine vector.

Subsequently, studies on generation of the NDV vector vaccines started to occur. In 2001, a recombinant NDV strain Hitchner B1 expressing the hemagglutinin (HA) gene of H1N1 influenza virus (A/WSN/33) was generated and this virus induced a strong antibody response against influenza virus and provided complete protection from the H1N1 challenge in mice [15]. This study for the first time confirmed immunogenicity and efficacy of NDV-vectored vaccines in animals. The first report on NDV-vectored vaccine in poultry was published two years later. Swayne et al. showed that an H7N2 avian influenza virus (AIV) vaccine based on NDV B1 strain stably expressed the HA protein, however, the NDV-H7N2 vaccine only provided 40% protection against the challenge with virulent NDV and highly pathogenic H7N2 virus in chickens [16]. The low protection against homologous NDV and AIV may be attributed to poor replication of the vector virus in the respiratory tract and the authors concluded that a booster immunization may be needed to enhance the efficacy of the vaccine. Nevertheless, their study still revealed the potential of NDV for use as a vaccine vector in its natural host. Currently, NDV-vectored influenza vaccines have been licensed in China and Mexico for use in the field. In 2006, a La Sota-based vaccine expressing the HA gene of H5N1 AIV was approved for use in chickens as a bivalent, live attenuated vaccine for controlling H5N1 avian influenza and Newcastle disease (ND) in China [17]. A total of four billion doses of this vaccine were applied in the first two years in chickens and the HA insert was kept updated with the evolvement of H5 AIV in China. In Mexico, the NDV vector vaccine with the H5N2 HA gene was used as a live and killed vaccine [18,19]. Commercialization of NDV-H5 vaccines is an encouraging event for NDV-vectored vaccines even for the field of viral vector vaccines.

However, although the NDV vector is considered as a promising vehicle, there has been a debate about the safety of this vector, i.e., the recombination risk between NDV vaccine strains and wild-type (wt) strains as well as between NDV and the host. In 2008, after several NDV-vectored vaccine candidates have been generated, Han et al. sounded the alarm for the safety of live vaccines based on the NDV vector by questioning whether the possibility of genetic exchange was thoroughly addressed involving NDV [20]. Based on the findings from their own and others, the authors concerned about that the recombination rate in NDV might not be low because a natural multirecombination in NDV and homologous recombination between live NDV vaccines and wt virus were identified [21,22]. In response, Collins et al. stated that the recombination is an infrequent event for NNSV and genetic exchange is not a practical concern for vaccinology [23]. Additionally, insertion of foreign genes in NNSV does not enhance the virulence and tropism of the virus but rather its attenuation. More importantly, Song et al. confirmed that the recombination of NDV is not as common as it was reported and that the identified recombination is likely artificial, resulting from sample contamination and mistakes in sequence assembly [24]. No studies reported on NDV recombination after these findings were published. Therefore, the report from Song et al. ended the debate by clarifying good safety of NDV as a vaccine vector in terms of low recombination risk.

Although the NDV vector vaccines are promising in preclinical studies, the interference of MDA is a major problem for the application of NDV-vectored vaccines in the poultry field. Almost all commercial chickens are vaccinated against NDV and chicks typically have high levels of MDA, which could impair the efficacy of NDV-vectored vaccines. In 2013, a novel approach of generating a chimeric vector by replacing the F and HN genes in NDV with those from antigenically-distinct avian paramyxovirus (APMV)-8 was reported [25]. The HA gene of H5N1 AIV was expressed in the chimeric NDV vector and the recombinant vaccine appeared to be highly immunogenic and efficacious in chicks with NDV MDA. This study provides a new method to overcome the interference of MDA. However, challenges related to this strategy still exist, and are discussed in details below.

Moreover, the insertion site of foreign genes in the NDV genome is a key issue for optimal gene expression and immunogenicity. NDV expresses its genes in a transcription gradient from 3′-NP-P-M-F-HN-L-5′. Correspondingly, a study showed that insertion of the CAT gene immediately before the 3′-promixal NP gene led to a high level of protein expression. However, no other insertion sites were analyzed in that study [26]. Zhao et al. compared expression levels of the secreted alkaline phosphatase (SEAP) inserted in different sites in NDV and found that gene insertion in the M-F junction led to the highest yield of SEAP [27]. Another report demonstrated that expression level of the H5 HA gene in different sites only differed moderately and genomic location of the HA gene between the F and HN genes resulted in the highest expression [28]. However, not all potential insertion sites in the NDV backbone were investigated in these two studies. In 2015, the green fluorescence protein (GFP) gene was inserted in five different sites of NDV (VG/GA strain) and the non-coding region between the P and M genes was identified as the optimal insertion site for foreign genes [29]. Consistently, the P-M junction was also determined as the optimal insertion site for the transgene in the APMV-3 backbone [30]. Currently, the P-M junction is used as the insertion site of foreign genes for most vaccines based on the NDV vector.

Expression of large-size foreign genes may be challenging due to the relatively low capacity of the NDV genome. Insertion of the transgene as an ITU attenuates transcription of downstream genes and virus replication. To overcome this drawback, different approaches for foreign gene expression in NDV have been explored, including expression of foreign genes in a two-segmented NDV genome and as an M-fusion protein linked by a self-cleavage 2A peptide [31,32]. However, there are concerns about the stability and antigenicity of foreign proteins for these approaches. In 2015, Zhang et al. expressed the red fluorescence protein (RFP) gene through an internal ribosomal entry site (IRES) as a second open reading frame in different locations in the NDV backbone [33]. Insertion of the IRES-RFP gene in different sites did not affect virus replication and red fluorescence intensity was positively correlated with the gene order of NDV. Subsequently, Hu and colleagues co-expressed the F gene of avian metapneumovirus (AMPV) under IRES and the glycoprotein (G) gene as an ITU in La Sota. The virus maintained similar virus yield and growth kinetics as the recombinant virus expressing the G gene and provided significant protection against AMPV-C challenge in turkeys [34]. They also co-expressed the RFP and GFP genes using the same strategy in NDV and the recombinant virus carrying two foreign genes exhibited similar in vitro characteristics when compared to the parental virus [35]. These studies indicate that the expression of multiple genes by a combination of IRES and ITU strategies may be a viable approach to generate multivalent vaccines or to express large-size genes in the NDV vector.

## 4. Recent Advances in the Development of NDV-vectored Vaccines and Therapeutics

Within two decades after NDV was explored as a vector, numerous vaccine candidates based on NDV have been generated for human and animal infectious diseases. Immunization and challenge studies in animal models demonstrated that these vaccine candidates are generally immunogenic and efficacious. Previously, four reviews have thoroughly summarized the development of NDV-vectored vaccines in human and veterinary medicine [8,9,36,37]. In the present article, an up-to-date list of vaccine and therapeutic candidates based on the NDV vector was included (Table 1).

For poultry, in addition to the conventional lentogenic NDV strains, modified live viruses (MLV) based on velogenic strains were also used as vaccine vectors. Some genotype VII NDV strains were attenuated by modifying the F cleavage site and used to express the immunogens from other poultry viruses [54,68,69,70]. Although these studies claimed the safety of these MLVs derived from virulent strains in preclinical experiments, caution should be taken when considering the safety of these MLV-based vaccines in poultry until they are thoroughly evaluated in the field. Furthermore, NDV-vectored vaccines have also been generated for waterfowls and game birds. Interestingly, the reported vaccines for waterfowls were all generated based on MLVs derived from genotype VII NDVs and the protective antigens of some emerging waterfowl viruses, such as duck Tembusu virus [70], goose parvovirus [69] and goose avastrovirus [68], were expressed. Compared to other genotypes, genotype VII NDVs are highly pathogenic in ducks and geese [98,99] and their attenuated derivatives may replicate well in waterfowls, which confer an advantage of genotype VII viruses as vectors for waterfowl vaccines. In addition, the Bornavirus vaccine based on NDV was generated and used as a prime component together with a modified vaccinia virus Ankara-vectored vaccine to elicit a robust immune response in cockatiels and common canaries [71,100], which further expands the species range of the NDV vector.

Recently, NDV was also used as a vector to generate novel vaccines for swine and cattle. The recombinant NDVs expressing the E2 and E^RNS^ genes of classical swine fever virus [76], the glycoprotein-3 and/or -5 genes of porcine reproductive and respiratory syndrome virus [77] and the glycoprotein gene of bovine ephemeral fever [72] were immunogenic in animals. As mentioned above, NDV can induce a systematic immune response in animals and acts as a hyperinducer of type I interferon (IFN), which endows this virus the potential as a vaccine vector as well as a cytokine adjuvant in mammalian animals.

On the other hand, some encouraging advances of the NDV vector in the field of human medicine have been also achieved recently. Two human vaccine candidates based on NDV against poliovirus and Japanese encephalitis virus were generated and these vaccines were immunogenic in animal models [91,95]. Particularly, the NDV-poliovirus vaccine produced virus-like particles in the cells of vaccine recipients and intranasal immunization of this vaccine induced strong mucosal and neutralizing antibody response in guinea pigs [91]. These studies, together with the previous reports, suggest that NDV is a promising vector to develop novel human vaccines. However, the NDV-vectored vaccines are currently evaluated in animal models and human trials are required to assess the safety, immunogenicity and efficacy of the vaccines. Of note, in addition to being used as a vaccine vector, two recent studies also explored NDV as a delivery vehicle of therapeutic antibodies against tumors. Vijayakumar and colleagues demonstrated that the recombinant NDV expressing the checkpoint inhibitor (anti-CTLA-4) worked together with radiotherapy to enhance tumor clearance of murine melanoma, indicating NDV is an effective immunotherapy agent capable of transgenic expression of single-chain variable fragment antibody in vivo to spare systematic exposure [96]. They also generated the recombinant NDVs expressing mAbs (PD1/PD-L1 antibody), superagonists (CD28) and immunocytokines (IL-12) and found that the viruses induced tumor control and survival benefits in a highly aggressive murine melanoma model [97]. These studies ingeniously combine two biological features of NDV, i.e., oncolytic activity and gene delivery capacity, together in immunotherapy for tumors, which further extends the range of clinical application of the NDV vector in human medicine.

## 5. MDA Interference: A Major Bottleneck for NDV-Vectored Vaccines

### 5.1. MDA: Friend and Foe

MDA (or the pre-existing antibody) against NDV has the opposite significance for vector vaccines in poultry and mammals. In poultry, MDA plays a critical role in protecting young chicks from clinical diseases. However, NDV-specific MDA impairs the efficacy of ND vaccines in poultry. Early studies demonstrated that efficacy of ND vaccines was poor in commercial chickens with MDA [101,102,103]. Additionally, ND vaccine Ulster 2C was less immunogenic in layers with MDA, and thus larger doses were required to induce sufficient protection [104]. In contrast, the NDV-specific pre-existing antibody is generally absent in mammalian animals and humans, which in turn become a benefit for the NDV vector in these hosts. Many efforts have been taken to overcome the interference of MDA in poultry and unfortunately there is still no effective solution for this problem.

MDA can hinder the immune response to vaccination with infectious bursal disease virus [105], NDV [104] and AIV vaccines in commercial poultry flocks [106,107,108]. Therefore, it can be speculated that NDV-specific MDA would undermine the efficacy of vaccines based on this virus. However, conclusions from the related studies seem to be controversial. Bertran et al. showed that MDA against NDV at the time of vaccination prevented induction of the immune response from a primary or booster NDV-H5 vaccine [109]. Similarly, Steglich et al. reported that NDV-H5 vaccine provided complete protection in specific-pathogen-free chickens without NDV-specific MDA, whereas this vaccine only provided 50% protection against H5N1 challenge in MDA-positive chickens [25]. Moreover, a high immunization dose of La Sota-vectored H5N2 vaccine was required to overcome MDA against NDV and AIV and to provide 100% protection of commercial broilers against infection with AIV and virulent NDV [19]. In contrast, La Sota-ILTV immunization in commercial broiler chickens revealed that high NDV MDA titers did not significantly interfere with the immunoresponse against NDV as well as the protective immunity against ILTV [110]. Although a controversy on MDA impact still exists and the mechanism of MDA interference is not well understood, it is generally accepted that anti-NDV MDA in commercial poultry interferes with the efficacy of NDV-vectored vaccines. It is important to note that in addition to vector-specific antibodies, MDA against the foreign antigen also exhibits a prominent inhibitory effect on vector vaccines because foreign proteins are usually incorporated into NDV virions [111,112]. A study showed that H5-specific MDA had an even stronger interference against a recombinant NDV-H5 vaccine compared to NDV MDA [112]. Pre-existing antibodies against both the vector and the foreign antigen may exert a combined interference against the NDV vector vaccines, which further complicates the problem.

### 5.2. Mechanisms and Hypotheses of Inhibition of Vaccination by MDA

**Figure 2:**
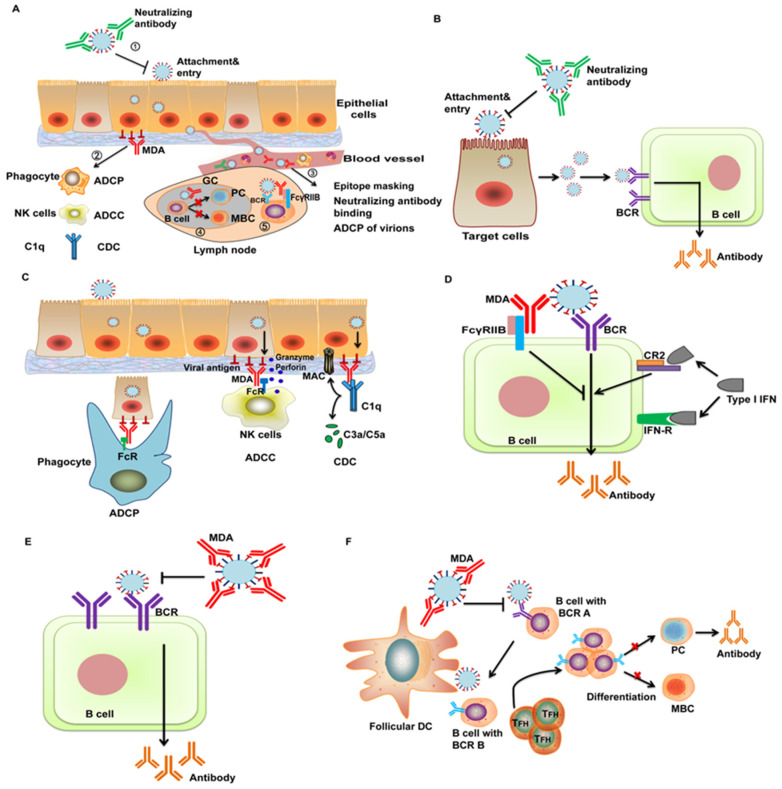
The mechanisms and hypotheses of vaccination inhibition by maternally derived antibody. (**A**) An overview of the impact of maternally derived antibody (MDA) on vaccination in different stages of immune response. (1) Neutralizing antibodies block the attachment and entry of the virus into cells. Epithelial cells are used here as an example of the vaccine recipient cells. (2) After virus replication and expression of the virus antigen on cell surface, MDA bridges virus-infected cells and effector cells, inducing immune effector functions, including antibody-dependent cellular cytotoxicity (ADCC), antibody-dependent cell-mediated phagocytosis (ADCP) and complement-dependent cytotoxicity (CDC), to clear virus-infected cells. (3) Upon entering the blood stream, the virus is recognized by MDA in the circulation, leading to epitope masking, neutralization and phagocytosis of the virus particles by phagocytes in the blood. (4) In lymph nodes, high titers of MDA modulate the differentiation of B cells into plasma cells (PC) and memory B cells (MBC) in the germinal centers. (5) Cross-linkage complex of the virus, B cell receptor (BCR) and FcγRIIB suppresses B cell activation. (**B**) Vaccination inhibition by neutralization. Binding of neutralizing antibodies to the virus blocks the attachment and entry of the vaccine virus and subsequently the virus replication required for antibody response. (**C**) Clearance of virus-infected cells by Fc-dependent effector functions. MDA binds to the viral antigen on the cells and antibody Fc domain binds to Fc receptors (FcR) on effector cells. Phagocytes engulf virus-infected cells (ADCP) and natural killer (NK) cells release granzyme and perforin to kill the cells (ADCC). IgG Fc binds to complement (C1q), leading to production of C3a/C5a and membrane attack complex (MAC) to eliminate infected cells (CDC). (**D**) Inhibition of antibody response by BCR-FcγRIIB cross-linkage. The inhibitory motif in FcγRIIB suppresses activation of B cells by cross-linking to antigen-specific BCR. Binding of type I interferon (IFN) to the IFN receptor (IFN-R) and complement receptor (CR) 2 provides a positive signal for B cell simulation. (**E**) Epitope masking. MDA binds to the immunodominant epitopes in the virus and thus blocks recognition of them by BCR. (**F**) Shaping B cell differentiation in the germinal centers. MDA recognizes the immunodominant epitopes and inhibits maturation of the B cells targeting these epitopes (B cells with BCR A). Maturation of the B cells targeting the non-immunodominant epitopes (B cells with BCR B) is enhanced but differentiation into PC and MBC is suppressed.

**Figure 3:**
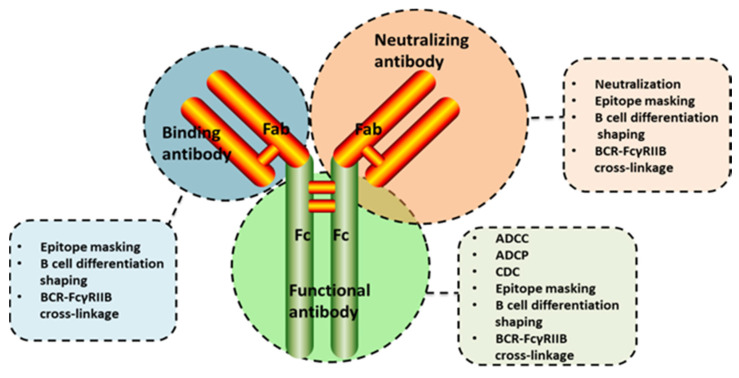
Possible mechanisms of vaccination inhibition by different classes of antibody. The functional roles of the antigen-binding domain (Fab) include recognizing viruses or virus-infected cells, mediating epitope masking, shaping of B cell differentiation in germinal centers and cross-linkage between B cell receptor and FcγRIIB. Additionally, antibodies targeting neutralizing epitopes induce virus neutralization and the interaction between the Fc domain and Fc receptors triggers antiviral effector functions, including antibody-dependent cellular cytotoxicity, antibody-dependent cell-mediated phagocytosis and complement-dependent cytotoxicity.

Although inhibition of vaccination by MDA has been well known in human and veterinary medicine for a long time, no effective vaccines or vaccination programs are developed because the mechanism is still obscure. Many studies have been carried out to elucidate the underlying mechanism in the field of human vaccinology and especially the interaction between measles vaccine (MV) and MDA has been thoroughly investigated. MDA interference with NDV-vectored vaccines is the biggest bottleneck for clinical application of the vaccines. However, no studies have been performed in poultry to elucidate the mechanism. Herein, the mechanisms of inhibition of vaccination by MDA based on the studies in mammalian animals and humans as well as some hypotheses will be discussed (Figure 2A and Figure 3). Meanwhile, whether these mechanisms and hypotheses would help to explain the interference with the NDV vector will be discussed and we also present our perspectives for future research directions and vaccine improvement.

#### 5.2.1. Virus Neutralization

An often-suggested explanation for inhibition of vaccination in the presence of MDA is the neutralization of the viral vector (Figure 2B). In theory, neutralizing MDA could impair replication of the vaccine by blocking attachment and entry of the virus into cells. Antibodies against the NDV vector can inhibit replication of the vaccine and the immune response. The heterologous prime-boost vaccination program using viral-vectored vaccines appears to be more efficacious compared to the homologous prime-boost scheme [44,113]. These findings suggest that pre-existing antibodies specific to the vector suppress the vector-vaccines. However, the contribution of neutralizing antibodies to vaccination inhibition still needs to be determined because non-neutralizing antibodies can also hamper vaccination with live attenuated vaccines [114]. Moreover, antibodies against foreign immunogens are also involved in inhibiting viral vectored-vaccines [115,116]. It has been shown that AIV-specific antibodies can inhibit seroconversion and protection of NDV-vectored AIV vaccines in commercial chickens, which is even stronger than inhibition from NDV-specific MDA [112]. In addition, AIV hyperimmunized serum passively transferred to chickens led to a significant reduction in the protective immune response to NDV-H5 vaccine [111]. DiNapoli et al. identified that recombinant NDV-H5 virus was insensitive to H5-specific neutralizing antibodies despite the HA protein being incorporated into virus particles [47], which argues against the inhibitory effect of neutralizing antibodies against the foreign antigen. However, the role of foreign antigens incorporated into NDV virions in the attachment and entry of the recombinant virus is still unclear. The current data is not sufficient to exclude the role of neutralizing antibodies against foreign antigens in vaccination inhibition. Thus, further studies are needed to characterize the role of vaccine antigens in the virus life cycle and to determine the impact of neutralizing antibodies against the vector and foreign antigens on NDV-vectored vaccines.

#### 5.2.2. Antibody-Dependent Effector Functions

In addition to neutralizing antibodies, antibodies that can mediate immune effector functions are also an important component in MDA, providing essential protection of neonates against infections. The F(ab)_2_ domain of non-neutralizing antibodies binds to the antigens and the Fc domain binds to the Fc receptors (FcR) on immune effector cells, such as natural killer cells, monocytes/macrophages and neutrophils. The cross-linkage among the antigen, antibody and the effector cells triggers immune effector functions, including antibody-dependent cellular cytotoxicity (ADCC), antibody-dependent cell-mediated phagocytosis (ADCP) and complement-dependent cytotoxicity (CDC), which contribute to the clearance of virus-infected cells and virus particles (ADCP particularly) (Figure 2C). Many sources of evidence have shown that effector functions mediated by antibodies are critical for protection against influenza virus, human immunodeficiency virus and respiratory syncytial virus [117,118,119]. In addition, ADCC antibodies are associated with the clinical presentation of neonatal herpes simplex virus (HSV) infection [120] and are protective against HSV-1 and -2 in a mouse model of maternal immunization [121]. Based on these findings, we can assume that effector functions mediated by MDA may reduce the replication and the antigen content after immunization with vector vaccines through elimination of virus-infected cells or virus particles. However, there is no direct evidence supporting this hypothesis. One possible reason is that characterizing the effect of functional antibodies and certain types of effector cells on immunization in vivo, especially in poultry, is difficult due to a lack of immunological reagents and standardized procedures. These studies are important to comprehensively understand the impact of different fractions of MDA on vaccination, which deserves more efforts.

#### 5.2.3. B Cell Inhibition by Cross-linking of BCR and FcγRIIB

B cell response is initiated by a three-signal cascade: the first signal, interaction of B cell receptor (BCR) with the antigen; the second signal, interaction with T cells through CD40-CD40 ligand; the third signal, soluble mediators, such as interleukin-6 and IFN-α. Interruption of any step could block B cell activation. There is a hypothesis that maternal antibody (IgG) simultaneously binds to FcγRIIB on B cells through the Fc domain and to BCR through the antigen binding domain, resulting in cross-linkage of BCR and FcγRIIB [122,123] (Figure 2D). The inhibitory motif in FcγRIIB counteracts the signaling of the activation motif of the BCR, leading to suppression of antigen-specific B cells by blocking the first signal for B cell activation. In vitro cross-linking of FcγRIIB with BCR potently inhibits B cell activation [124,125] and FcγRII-deficient mice show enhanced antibody response compared to wt mice [126,127]. Kim et al. found that MV-specific IgG, rather than the F(ab)_2_ fragments, and a mAb with FcγRIIB non-binding Fc domain, can inhibit both B cell proliferation and antibody production [114]. Their results verified the BCR-FcγRIIB cross-linkage hypothesis using a measles vaccination model in vitro and in vivo. However, the findings that IgG-mediated inhibition of antibody response is comparable in FcγRIIB-deficient mice and wt mice argue against this hypothesis [128,129]. Biologically, pre-existing antibodies in the body may give feedback to the immune system to avoid over-reactive B cell response, but the available data on the role of FcγRIIB-BCR cross-linking seems to be conflicting, which may be explained by different antigens and animal models used in these studies.

#### 5.2.4. Epitope Masking

Another hypothesis for the lack of protective B cell responses in the presence of MDA is epitope masking [122,130] (Figure 2E). This idea postulates that the B cell epitopes that trigger B cell response in the antigen are targeted and covered by MDA in the circulation, which will block binding of the antigen with specific BCR on B cells. This hypothesis is supported by many studies using antibody feedback models [130,131,132]. IgG binding with low-density epitopes in the antigen caused epitope-specific suppression, and binding with high-density epitopes led to non-epitope-specific inhibition through steric hindrance with adjacent epitopes [130]. In addition, different isotypes and F(ab)_2_ fragments can also suppress antibody response by epitope masking [133,134]. However, Kim et al. showed that only complete IgG instead of the F(ab)_2_ fragments can block MV vaccination in cotton rats, suggesting that Fc-region is required for inhibition of antibody generation [114]. Additionally, they also demonstrated that a mAb targeting single epitope can also suppress recognition of the antigen by B cells, indicating that B cell inhibition is not epitope-specific. The observations from Kim et al. favor the BCR-FcγRIIB cross-linking mechanism but contradict the epitope masking hypothesis, which may be associated with different experimental models and antigens used in these studies.

#### 5.2.5. Shaping the Early-Life B Cell Repertoire in Germinal Centers by MDA

Importantly, a recent study discovered a novel and sophisticated mechanism of vaccination inhibition by MDA [135]. They developed well-designed neonatal/infant immunization models and showed that MDA does not prevent B cell activation or germinal center (GC) formation but controls plasma cell (PC) and memory B cell (MBC) differentiation quantitatively and qualitatively, thus shaping the long-term antigen-specific B cell repertoire (Figure 2F). High titers of MDA significantly depressed neonatal antibody response to vaccination with influenza HA antigen. Vaccination in the presence of MDA did not affect CD4+ T effector cell responses but caused the premature decline of T follicular helper cells, which is essential for B cell maturation. Additionally, even high titers of MDA did not prevent B cell activation, B cell differentiation in GC and the bona fide GC responses to neonatal immunization. Nevertheless, MDA drastically manipulated the GC output by reducing or blocking the induction of PCs and MBCs in a MDA- and antigen dose-dependent manner. The underlying mechanism for these phenomena may be that MDA hijacks the antigen required for the affinity maturation process of B cells by binding to the immunodominant epitopes and in turn forces binding of early-life B cells to non-immunodominant epitopes, which may limit their recruitment into the GCs and survival through the GC selection and PC differentiation process. Of note, their observations do not exclude the roles of epitope masking or of BCR-FcγRIIB cross-linkage in inhibiting B cell activation. Instead, they may represent different mechanisms co-existing in the process of inhibition of vaccination by MDA.

## 6. Efforts to Circumvent the Interference of MDA with the NDV Vector

Many efforts have been taken to overcome MDA interference, including application of higher doses, more aggressive vaccine strains and multiple immunizations. For ND or NDV-vectored vaccines, three main strategies were used to overcome or sidestep the interference of MDA.

### 6.1. Generation of Chimeric NDV Vectors

A novel approach to the NDV vector engineering becomes attractive. APMVs are classified into sixteen serotypes (APMV-1 to -15 and APMV-17) based on their antigenicity [136] and they have similar genomic structures. Previous studies showed that NDV (APMV-1) has low cross-reactivity with other APMVs except for APMV-3 [137]. Thus, these features provide an opportunity to construct chimeric NDV vectors by replacing the main antigens (F and HN) in NDV with the counterparts from other APMVs. Different groups have generated vaccines based on such vectors and evaluated their immunogenicity and efficacy in the presence of NDV MDA. Steglich et al. generated a chimeric NDV-APMV8 vector and expressed the H5N1 HA gene in this backbone [25]. The vaccine based on this chimeric vector provided solid protection against lethal AIV infection in chickens with NDV MDA. Similarly, two independent teams constructed chimeric NDV-APMV2 viruses expressing the HA genes of H5N1 or H9N2 AIVs and the vaccines stimulated protection against AIV infection in the presence of anti-NDV pre-existing immunity [43,50]. Therefore, the current findings suggest that vaccines based on chimeric NDV vectors can circumvent the interference from NDV-specific MDA and have the potential to be used in commercial poultry.

All the mechanisms or hypotheses for vaccination inhibition by MDA described above may be applicable for explaining successful induction of antibody response by chimeric APMV vectors in the presence of MDA. Replacement of the major antigens of NDV may result in escape from binding to MDA specific to NDV. However, the exact mechanism for inhibition of NDV-vectored vaccines by MDA and how chimeric NDV vectors circumvent MDA interference remains to be determined.

Although chimeric NDV vectors seem to be effective in the presence of NDV MDA, there are still three concerns about this strategy. First, circumventing MDA interference in this way is achieved at the price of sacrificing the protection against NDV per se. In this case, additional vaccination against NDV may be needed to eliminate the lack of the protection from virulent NDV, which may increase the cost of vaccination [138]. Although ND vaccination is routinely performed in poultry flocks, the aim of ideal vaccination with virus vectors is to induce potent protective immunity against multiple diseases. NDV-vectored vaccines that can provide dual protection would be more efficient and cost-effective. Secondly, the vaccines based on chimeric vectors cannot overcome the interference from anti-transgene immunity. Our group demonstrated that H9N2 vaccine based on NDV-APMV2 chimeric vector elicited higher HI titers against H9N2 in NDV MDA chickens compared to that in NDV + AIV MDA chickens (unpublished data), indicating a robust interference with the chimeric vector vaccine by MDA against the foreign antigen. Last, the presence of APMV antibodies (other than NDV) in commercial poultry may undermine the efficacy of chimeric NDV vectors. APMV-2 to -15 and APMV-17 were mainly isolated from a variety of wild birds worldwide and APMV transmission between wild birds and domestic poultry was reported [139,140]. A serosurveillance of commercial poultry farms in USA showed the presence of antibodies to APMV-1 to -9 except for APMV-5 [141], which may interfere with the effectiveness of chimeric APMV vectors. However, evidence of seroprevalence of APMV in commercial poultry is rare and more seroepidemiology studies are required. Therefore, chimeric NDV vectors may not be the only and best approach to circumvent the interference of MDA. Understanding the underlying mechanism of MDA interference is greatly needed, which is the basis to improve NDV-vectored vaccines resistant to MDA.

### 6.2. Restoration of B Cell Response by Cytokine Stimulation

Induction of cytokines serving as the third signal is another feasible way to reactivate B cell response in the presence of MDA. Complement receptor (CR) 2 on B cells binds C3d complement and IFN-α with the same affinity [142] and stimulation with IFN-α upregulates B cell genes in a B cell line [143]. Binding of CR2 with IFN-α acts as a stimulatory signal for antibody production (Figure 2D). MV cannot induce IFN-α in dendritic cells in vitro nor in cotton rats, whereas a recombinant NDV expressing the MV H protein (NDV-H) induced potent IFN-α secretion [144]. In NDV-H-immunized cotton rats, neutralizing antibody response was partially restored in the presence of measles-specific MDA, indicating the role of IFN-α as an additional signal for antibody induction [144]. However, NDV is also a hyperinducer of IFN-α in its natural host chickens, whereas the enhancement of antibody response by NDV-vectored vaccines in the presence of MDA was not seen. Thus, the effect of type I IFN on antibody immunity against NDV-vectored vaccines needs to be determined in chickens.

More importantly, Zhang et al. reported that the recombinant NDVs expressing granulocyte-macrophage colony-stimulating factor (GM-CSF) or flagellin (fliC) genes can induce a quick immune response and are resistant to MDA in chickens [145]. The two recombinant viruses induced higher HI titers against NDV in MDA-positive chickens compared to the vector virus. GM-CSF or fliC expressed in NDV may enhance T cell maturation, particularly CD4+ T cells, which contribute to B cell activation and antibody response. Their study represents a different mechanism of B cell activation, which, together with MV studies, suggests a critical role of cytokines in restoration of B cell immunity in the presence of MDA. However, it is still interesting to see how NDV co-expressing cytokine and protective antigen may behave in the presence of MDA.

### 6.3. Overcoming MDA Interference by Increasing Antigen Dose

High titers of MDA, hijacking a lot of vaccine antigens, may leave a small fraction of antigens for affinity maturation and differentiation of B cells. It is feasible to apply sufficiently high enough antigen doses in vaccines when maternal immunization is conducted. Such practice has been implemented for vaccination against respiratory syncytial virus in children. Similarly, an Ulster 2C-derived NDV vaccine was less immunogenic in layers with MDA, thus requiring large doses (≥ 7.0 log_10_ EID_50_) to induce sufficient protection [104]. In addition, Sarfati-Mizrahi et al. demonstrated that 6.8 log_10_ EID_50_ or higher doses of NDV-H5N2 vaccine were required to stimulate complete protection against AIV challenge in the presence of MDA against NDV and AIV [19]. However, from the economic viewpoint, a vaccine dose per chicken of ≥ 7.0 log_10_ EID_50_ is not practical.

## 7. Conclusions

During the past two decades, a variety of foreign antigens have been expressed in the NDV vector and the strategy of foreign gene expression in NDV has been optimized. These substantial advances strengthen the potential of NDV as a vaccine vector for veterinary and human use.

## 8. Perspectives

For poultry, to enhance the effectiveness of NDV-vectored vaccines in the field, it is vital to find a way to circumvent the interference of MDA. The first priority to achieve this goal is to elucidate the underlying mechanism of inhibition of vaccination by MDA specific to the vector as well as the foreign antigen, which is the basis to improve the vaccines. We propose some research topics deserving more attention: the effect of MDA on B cell activation and differentiation, the contribution of neutralizing as well as effector functional antibodies to vaccination suppression, T cell immunity against NDV-vector vaccines and its role in B cell response in the presence of MDA and the role of cytokines in B cell stimulation. Meanwhile, based on the available data and hypotheses, it is still reasonable to test the effect of cytokines on antibody response in the presence of MDA by generating NDVs co-expressing the cytokine and foreign genes. In addition, further enhancement of vector replication in the presence of MDA is an alternative to present more antigens for B cell maturation and differentiation.

Conversely, due to a lack of pre-existing NDV-specific antibodies and the ability of NDV to stimulate IFN-α, the NDV vector vaccines can serve as an option for immunization against important infectious diseases in mammals and humans. Particularly, in the case of emerging infectious disease in humans, such as H7N9 avian influenza and COVID-19, NDV-vectored vaccines can serve as an alternative for emergency vaccination because generation of recombinant NDVs in laboratories is fast and immunization via eyedrops or intranasal spray is convenient and time-saving.

All the research work in the past 20 years has proved the potential of NDV as a vaccine vector and has unmasked an unsolved problem as well as the future direction. More progress and substantiation of the contribution of the NDV vector to human and veterinary medicine are in prospect in the next 20 years.

## Figures and Tables

**Table 1:** Vaccine and therapeutic candidates based on the Newcastle disease virus (NDV) vector for veterinary and human medicine.

Pathogen/Disease	Antigen	NDV Backbone	Animal Model	Route of Immunization	Dose	Reference
H1N1	HA	Hitchner B1	mouse	i.v. or i.p.	two doses, 5 × 10^7^ pfu	[15]
H5N1	HA	La Sota	chicken/mouse	o.n.(chicken) i.p.(mouse)	one dose, 10^6^ EID_50_ (chicken); two doses, 10^6^ EID_50_ (mouse)	[38]
H5N1	HA	La Sota	chicken	o.n.	one dose, 10^6^ EID_50_	[39]
H5N2	HA	La Sota	chicken	i.m./spray	two doses, 5 × 10^6^ TCID_50_ (i.m.); one dose, 10^6^ TCID_50_ (spray)	[40]
H5N1	HA	La Sota	chicken	i.m. or o.n.	two doses, 5 × 10^6^ pfu	[41]
H5N2	HA/HA+NA	La Sota/ chiNDV-2FHN	chicken	i.n.	prime, chimeric-vector vaccines, 10^5^ pfu; boost, La Sota-vector vaccines, 10^5^ pfu	[42]
H5N1	HA	chiNDV-8FHN	chicken	o.n.	one dose, 10^6^ TCID_50_	[25]
H5N1	HA	chiNDV-2FHN	chicken	o.n.	two doses, 10^6^ pfu/mL	[43]
H5N1	HA or HA + NA/M1/NS1	La Sota/ chiNDV-2FHN	chicken	o.n.	one dose, 10^6^ pfu/mL; two doses, chimeric-vector prime and La Sota-vector boost, 10^6^ pfu/mL	[44]
H5N1	HA/HA1	TS09-C	chicken	i.n./i.o.	two doses, 10^6^ TCID_50_	[45]
H5N1	HA	La Sota	duck	i.o.	two doses, 10^6^ EID_50_	[46]
H5N2	HA	La Sota	chicken	i.o.	one dose, 10^4.8^, 10^5.8^, 10^6.8^, 10^7.8^EID_50_	[19]
H5N1	HA	BC	monkey	i.n./i.t.	two doses, 10^7^ pfu	[47]
H9N2	HA	La Sota	chicken	o.n./i.m.	two doses, 10^7^ ffu	[48]
H9N2	HA	NA strain	chicken	o.n.	one or two dose, 10^6^ EID_50_	[49]
H9N2	HA	chiNDV-2FHN	chicken	o.n.	one dose, 10^6^ EID_50_	[50]
H7N2	HA	Hitchner B1	chicken	i.o.	one or two dose, 10^5.7-6.1^ EID_50_	[51]
H7N2	HA	Hitchner B1	chicken	i.o.	one dose, 10^6^ EID_50_	[16]
H7N1	HA	La Sota	chicken	i.n.	one dose, 10^6^ EID_50_	[52]
H7N9	HA	La Sota	chicken	i.m. or o.n.	two doses, 5 × 10^6^ pfu	[41]
H7N9	HA	LX	chicken	i.n.	two doses, 5 × 10^6^ EID_50_	[53]
H7N9	HA	rAI4	chicken	i.n./i.o.	one dose, 10^6^ EID_50_	[54]
H7N3 H7N8	HA HA/HA + NA	La Sota La Sota/ chiNDV-2FHN	mouse chicken	i.n. i.n.	two doses, 10^4^ or 10^6^ ffu prime, chimeric-vector vaccines, 10^5^ pfu; boost, La Sota-vector vaccines, 5 × 10^5^ pfu	[55] [56]
H6N2	HA	Clone 30	chicken/turkey	o.n.	one dose, 10^6^ (chicken)/10^7^ (turkey)EID_50_	[57]
IBDV IBDV	VP2 VP2	La Sota F	chicken chicken	i.o.	one or two dose, 10^4^ ELD_50_ two dose, 10^6^ EID_50_	[58] [59]
IBDV	VP2	rLaC30L	chicken embryo	in ovo	one dose, 10^5.5^, 10^4.5^, 10^3.5^, 10^2.5^EID_50_	[60]
ILTV	gB/gD	La Sota	chicken	i.n./i.o.	one dose, 10^6^ TCID_50_	[61]
ILTV	gB/gC/gD	La Sota	chicken	o.n.	two doses, 2 × 10^5^ TCID_50_	[62]
IBV	S	La Sota	chicken	o.n.	one or two dose, 10^6^ pfu	[63]
IBV	S1	La Sota	chicken	o.n.	one or two dose, 10^6^ pfu	[64]
IBV	S1 (multi-epitope)	La Sota	chicken	o.n.	one dose, 10^6^ EID_50_	[65]
AMPV	G	La Sota	turkey	i.n./i.o.	one or two dose, 10^6^ TCID_50_	[66]
AMPV	F+G	La Sota	turkey	i.n./i.o.	one dose, 10^6^ TCID_50_	[34]
FAdV	fiber 2	La Sota	chicken	i.m.	one dose, 10^7^ EID_50_	[67]
GoAstV	Cap	SH12	gosling	o.n.	one dose, 10^7^ TCID_50_	[68]
GPV	VP3	NA	gosling	s.c.	two doses, 10^6^ EID_50_	[69]
DTMUV	prM+E	GM	duck	s.c.	two doses, 10^6^ EID_50_	[70]
Bornavirus	N/P	Clone 30	cockatiel/canary	i.m.	10^5.9-6.1^ (cockatiel)/10^6.6^ ffu (canary)	[71]
BEFV	G	La Sota	cattle	i.m.	two doses, 8 × 10^7^ EID_50_	[72]
BHV-1	gD	La Sota	calf	i.n./i.t.	one dose, 1.5 × 10^7^ pfu	[73]
CDV	F/H	La Sota	mink	i.m.	two doses, 2 × 10^9^ EID_50_	[74]
Rabies	G	La Sota	dog	i.m.	three doses, 10^9.8^/10^9.3^/10^8.3^ EID_50_	[75]
CSFV	E2/E^rns^	La Sota	pig	i.n.	two doses, 10^3^ TCID_50_	[76]
PRRSV	GP5/GP3 + GP5	La Sota	piglet	i.m.	two doses, 4 × 10^8^ EID_50_	[77]
VSV	G	La Sota	mouse	i.m.	two doses, 10^7^ TCID_50_	[78]
HIV-1	Gag	Hitchner B1	mouse	i.n.	prime, 5 × 10^5^ pfu; boost, 10^6^ pfu	[79]
HIV-1	Gag	La Sota	mouse	i.n.	prime, 5 × 10^5^ ffu; boost, 10^6^ ffu	[80]
HIV-1	Gag/Env/ Gag + Env	La Sota	guinea pig/mouse	i.n.	two doses, 2 × 10^5^ (guinea pig)/4 × 10^3^ (mouse) pfu	[81]
SIV	gp160	La Sota/ chiNDV-2FHN	guinea pig	i.n.	two doses, 10^5^ TCID_50_	[82]
EBOV	GP	BC/La Sota	monkey	i.n./i.t.	two doses, 10^7^ pfu	[83]
EBOV	GP	APMV-3/ chiNDV-3FHN	guinea pig	i.n.	two doses, 2 × 10^6^ TCID_50_	[84]
HPIV-3	HN	BC	monkey	i.n./i.t.	two doses, 10^6.5^ pfu	[85]
NiV	G/F	La Sota	pig	i.m.	two doses, 2 × 10^9^ EID_50_	[86]
NV	VP1 + VP2	BC/La Sota	mouse	i.n.	three doses, 10^6^ EID_50_	[87]
SARS-CoV	S	BC/La Sota	monkey	i.n./i.t.	two doses, 10^7^ pfu	[88]
MERS-CoV	S	La Sota	mouse/camel	i.m.	two doses, 10^8^ (mouse)/ 2 × 10^9^(camel) EID_50_	[89]
RSV	F	Hitchner B1	mouse	i.n.	one dose, 5 × 10^5^ pfu	[90]
poliovirus	P1 + 3CD	La Sota	guinea pig	i.n.	two doses, 10^5^ pfu	[91]
Lyme	BmpA/OspC	La Sota	hamster	i.n./i.m./i.p.	two doses, 10^6^ pfu	[92]
RVFV	Gn	La Sota	cattle	i.n. /i.m.	two doses, 10^6.3^ (i.n.)/ 2 × 10^7^ (i.m.) TCID_50_	[93]
WNV	PrM/E	La Sota	mouse/house	i.m.	two doses, 10^8^ (mouse)/ 2 × 10^9^ (horse) EID_50_	[94]
JEV	E/NS1	La Sota	mouse	i.n.	one dose, 10^6^ EID_50_	[95]
melanoma	PD1/PD-L1/CTLA4	La Sota	mouse	i.t	five injections, 1 × 10^7^ pfu	[96]
melanoma	PD1/PD-L1/CD28	La Sota	mouse	i.t	five injections, 1 × 10^6^ pfu	[97]

IBDV, infectious bursal disease virus; ILTV, infectious laryngotracheitis virus; IBV, infectious bronchitis virus; AMPV, avian metapneumovirus; FAdV, fowl adenovirus; GoAstV, goose origin avastrovirus; GPV, goose parvovirus; DTMUV, duck tembusu virus; BEFV, bovine ephemeral fever virus; BHV-1, bovine herpesvirus-1; CDV, canine distemper virus; CSFV, classical swine fever virus; PRRSV, porcine reproductive and respiratory syndrome virus; VSV, vesicular stomatitis virus; HIV-1, human immunodeficiency virus-1; SIV, simian immunodeficiency virus; EBOV, Ebola virus; HPIV-3, human parainfluenza virus type-3; NiV, Nipah virus; NV, norwalk virus; SARS-CoV, severe acute respiratory syndrome-associated coronavirus; MERS-CoV, Middle East respiratory syndrome coronavirus; RSV, respiratory syncytial virus; RVFV, Rift Valley fever virus; WNV, West Nile virus; JEV, Japanese encephalitis virus; HA/H/HN, hemagglutinin; NA, neuraminidase; M1, matrix 1; NS1, non-structural protein 1; HA1, hemagglutinin subunit 1; VP1/2/3, viral protein 1/2/3; gB/gC/gD, glycoprotein B/C/D; S, spike; S1, spike subunit 1; G, glycoprotein; F, fusion; Cap, capsid protein; prM, pre-membrane protein; E, envelop protein; N, nucleoprotein; P, phosphoprotein; E2/E^rns^, envelop glycoproteins; GP3/5, glycoprotein 3/5; P1, capsid protein precursor; 3CD, viral protease; PD1, programmed cell death-1; PD-L1, programmed cell death 1 ligand 1; CTLA4, cytotoxic T-lymphocyte-associated antigen-4; APMV, avian paramyxovirus; chiNDV-2FHN, chimeric NDV-APMV 2 virus; chiNDV-8FHN, chimeric NDV-APMV 8 virus; chiNDV-3FHN, chimeric NDV-APMV 3 virus; i.v., intravenous; i.p., intraperitoneal; o.n., oculonasal; i.m., intramuscular; i.n., intranasal; i.o., intraocular; i.t., intratumoral; s.c., subcutaneous; pfu, plaque forming unit; EID_50_, 50% embryo infectious dose; TCID_50_, 50% tissue culture infectious dose; ffu, focus forming unit; ELD_50_, 50% embryo lethal dose.
